# Grammar Is Differentially Impaired in Subgroups of Autism Spectrum Disorders: Evidence from an Investigation of Tense Marking and Morphosyntax

**DOI:** 10.3389/fpsyg.2017.00320

**Published:** 2017-03-28

**Authors:** Nadezhda Modyanova, Alexandra Perovic, Ken Wexler

**Affiliations:** ^1^INTERLINK Language Center, Montana State UniversityBozeman, MT, USA; ^2^Department of Linguistics, Psychology and Language Sciences, University College LondonLondon, UK; ^3^Department of Brain and Cognitive Sciences and Department of Linguistics and Philosophy, Massachusetts Institute of TechnologyCambridge, MA, USA

**Keywords:** autism, language development, language impairment, verbal inflection, optional infinitives, finiteness, morphosyntax

## Abstract

Deficits in the production of verbal inflection (tense marking, or finiteness) are part of the *Optional Infinitive* (OI) stage of typical grammatical development. They are also a hallmark of language impairment: they have been used as biomarkers in guiding genetic studies of Specific Language Impairment (SLI), and have also been observed in autism spectrum disorders (ASD). To determine the detailed nature of finiteness abilities in subgroups of ASD [autism with impaired language (ALI) vs. autism with normal language (ALN)], we compared tense marking abilities in 46 children with ALI and 37 children with ALN with that of two groups of nonverbal mental age (MA) and verbal MA-matched typically developing (TD) controls, the first such study described in the literature. Our participants' performance on two elicited production tasks, probing third-person-singular -*s* and past tense -*ed*, from the *Rice/Wexler Test of Early Grammatical Impairment* (TEGI, Rice and Wexler, 2001), revealed extensive deficits in the ALI group: their ability to correctly mark tense was significantly worse than their much younger TD controls', and significantly worse than that of the ALN group. In contrast, the ALN group performed similarly to their TD controls. We found good knowledge of the meaning of tense, and of case and agreement, in both ASD groups. Similarly, both ASD groups showed distributions of null or overt subjects with nonfinite and finite verbs in line with those found in young TD children. A key difference, however, was that the ALI group used (rather than simply omitted) the wrong tense in some sentences, a feature not reported in the OI stage for TD or SLI children. Our results confirm a clear distinction in the morphosyntactic abilities of the two subgroups of children with ASD: the language system responsible for finiteness in the ALN group seems to be functioning comparably to that of the TD children, whereas the ALI group, despite showing knowledge of case and agreement, seems to experience an extensive grammatical deficit with respect to finiteness which does not seem to improve with age. Crucially, our ALI group seems to have worse grammatical abilities even than those reported for SLI.

## Introduction^1^

One key difference between adult and child grammar, according to Wexler (e.g., 1994, 1998, 2003, 2004a, 2011), is that for typically developing (TD) children of a certain age, sentences may optionally be finite (with tense markers) or nonfinite (with infinitival form of the verb), hence the name Optional Infinitive (OI) Stage. Typical errors are illustrated below, where the child omits the present tense inflection -*s* with a regular verb in (1), or produces the actual infinitive form of an irregular verb, as in (2).

(1) He bite my fingers. (Nina 2;0.0, CHILDES, Pierce, 1992:99)
(2) Her have a big mouth. (Nina 2;2.6, CHILDES, MacWhinney, 2000)

At the same time that TD children produce OIs, they demonstrate knowledge of the difference between nonfinite and finite verbs, as well as other important aspects of morphosyntactic structure (see Wexler, 1994, and Guasti, 2017, for crosslinguistic evidence). For example, in English, children's nonfinite verbs often occur with default case subject pronouns (accusative in English), and finite verbs predominantly appear with nominative case pronouns (Schütze and Wexler, 1996). Children of this age also know subject-verb agreement; they nearly always produce a third person singular subject when *-s* appears on the verb (e.g., Rice et al., 1995; Harris and Wexler, 1996). Children also do not misuse tense (i.e., they do not use present in the past tense context, and vice versa) (Rice et al., 1995; Schütze and Wexler, 2000). Finally, young children produce null subjects (i.e., they omit overt subjects from their sentences), and they do so more often with nonfinite verbs than with finite verbs (see Hyams, 2011; Wexler, 2013).

Difficulties with finiteness have been observed in other developmental disorders, most notably in Specific Language Impairment (SLI). Children with SLI are found to be considerably delayed in finiteness relative to both their TD language- and age- matched controls, and this phenomenon was termed the Extended Optional Infinitive (EOI) stage (Rice et al., 1995, 1998, 1999, 2000, 2009a; Rice and Wexler, 1996; Wexler, 1996; Wexler et al., 1998; among others). The work on EOI culminated in the creation of a diagnostic elicited production test, the Rice/Wexler Test of Early Grammatical Impairment (TEGI, Rice and Wexler, 2001), which we use in the present study.

Our paper addresses whether children with ASD show the pattern of morphosyntactic phenomena associated with the OI stage in TD and with the EOI stage in SLI so that we can answer whether children with ASD are in the EOI stage. This topic is particularly interesting in view of the current controversies on whether similarities in linguistic profiles of SLI and ASD are indicative of links between these two populations. Some researchers contend that patterns of linguistic impairments in ASD are reminiscent of those in SLI (e.g., Kjelgaard and Tager-Flusberg, 2001; Lindgren et al., 2009) and that the two populations may be on a continuum. However, others argue that the similarities in linguistic profiles of the two populations are only superficial (e.g., Bishop, 2003a; Whitehouse et al., 2008). To make the matter more complicated, the literature is not in agreement on whether grammatical morphology, and especially finiteness, is impaired in ASD at all. Early work on grammatical morphology in ASD reports deficits in the use of correct verbal inflection in spontaneous speech (Bartolucci et al., 1980), later confirmed experimentally by Roberts et al. (2004). However, more recent work, focusing on higher functioning children with ASD, reports little if any difficulty in this domain (Eigsti and Bennetto, 2009; Walenski et al., 2014). Crucially, the population with ASD is known for extreme heterogeneity in language and cognitive skills; thus, different patterns may be observed in children who are higher functioning in terms of language and nonverbal reasoning, vs. those who are at the lower end of the spectrum in these domains.

To establish whether there exists a deficit in finiteness in ASD, similar to that observed in SLI, we carried out an experimental study employing materials used to establish finiteness difficulties in SLI, in a large number of children with ASD of heterogeneous abilities, divided into two groups in line with the classifications in the literature (Kjelgaard and Tager-Flusberg, 2001): those with relatively spared language (“autism language normal,” ALN) and those with an established language impairment (“autism language impaired,” ALI). To presage our results, children with ALN will show evidence that they are outside of the OI stage; in fact, their nonfiniteness levels will be not much higher than their TD controls'. On the other hand, children with ALI will not only show very low finiteness rates and some properties typical of the OI stage, but they will also show some other properties that are quite inconsistent with the OI stage, representing a deviant and disrupted grammar. The most striking example will be the large number of errors with using the wrong tense, a property not found in TD or SLI children. These results will suggest that children with ALI are not simply children with ASD who also have SLI.

### Finiteness in ASD

Considering how well researched finiteness is in TD and in SLI, and especially the current debates of possible links between SLI and ASD (e.g., Tager-Flusberg, 2015), it is surprising how little research there exists on this topic in ASD.

Early studies focusing on grammatical morphology in spontaneous speech in children with ASD report difficulties with both past and present tense; however, results from these studies, which included small samples of children with autism and with heterogeneous language and cognitive abilities, are far from clear. Bartolucci et al. (1980) report that 10 ten-year-old boys with impaired nonverbal IQ (NVIQ) marked present tense correctly 86% of the time, irregular past tense 94% of the time, but regular past tense only 77% of the time. Somewhat higher performance is reported by Howlin (1984) in a sample of 16 autistic 8-year-old boys with normal NVIQ but delayed language: they marked present tense correctly 85% of the time, regular past tense at 84%, and irregular past tense at 97%. Only Bartolucci et al. (1980) included TD controls [matched on nonverbal mental age (MA)]; Howlin (1984) did not.

More recent studies used methods more akin to those used in SLI research, such as TEGI-type tasks which employ constrained elicited production of present and past tenses, rather than spontaneous speech. Botting and Conti-Ramsden (2003) compared past tense in 29 ten-year-olds with SLI and 13 age-matched children with ASD with borderline or normal NVIQ but heterogeneous language skills. An equally poor performance on past tense was reported in both populations; however, no details of differences on regular vs. irregular verbs were given, making finiteness rates impossible to determine.

In the only study that divides children with ASD into subgroups according to impaired or unimpaired language, as measured by vocabulary skills, Roberts et al. (2004) report high rates of tense marking in children with normal language and normal NVIQ (*n* = 27, 86% correct on composite past and 81% on present tense), somewhat worse performance in the group of children with “borderline” language scores (*n* = 16, 86% correct on past and 69% on present tense), and the worst performance in the group with impaired language abilities and borderline NVIQ (*n* = 19, 68% correct on past and 65% correct on present tense). This study did not have any control participants.

Studies using different methodologies still show subtle differences in the mastery of finiteness in ASD compared to control children. Eigsti and Bennetto (2009), using a grammaticality judgment task, report a relatively good performance in 21 high-functioning children with ASD aged 9–17 years (with high VIQ, NVIQ, and vocabulary scores). However, their performance on present and past tense was still worse compared to TD controls matched on a range of variables, such as age, nonverbal and verbal IQ, vocabulary, gender, and socioeconomic status.

The only study to show an age appropriate performance by children with ASD is Walenski et al. (2014). 20 ten-year-old high functioning boys with ASD, with normal nonverbal and verbal IQ and reading levels, showed performance of 96% correct for regular past tense and 64% overall for irregular past tense form (with 23% over-regularization rates), which was comparable to their age, IQ, and reading level-matched TD controls.

Taken together, these findings demonstrate a wide range of finiteness abilities for children with ASD. A clear trend is that children with ASD show finiteness performance below their chronological age level, just like children with SLI. Compared to TD controls (matched at least on NVIQ for all studies that used them), children with ASD are usually worse on tense marking, but this largely depends on whether ASD participants have higher or lower NVIQ levels.

### The present study

A major aim of the present study is to compare the production of finiteness in children with ASD to that of TD children functioning at similar nonverbal MA level. This approach allows us to characterize precisely the severity of the deficit in finiteness found in children with ASD relative to TD peers. Furthermore, we aim to infer whether the language system underlying finiteness in ASD is intact, delayed (showing similar patterns as younger matched TD controls), deviant (showing patterns not found in TD at all) or disrupted (showing worse performance than the youngest TD controls, that is a severe delay, suggesting an “asynchrony” in development, as is the case for children with SLI) (Rice, 2007:416). Thirdly, we aim to distinguish our results depending on whether the children with ASD are classified on the basis of their general language skills into those who have normal language (ALN) or those with impaired language (ALI).

In our study, the children with ALI have not only a deficit in general language ability but also nonverbal IQ deficits. The question we will want to ask is whether any delay that these children show on finiteness is due solely to their lower IQ and their lower level of general language abilities, or whether it goes beyond these. Our hypotheses are illustrated in (3).

(3) Hypotheses: Children with ALI may show:
  - Intact behavior on finiteness (equivalent to TD peers of the same age);
  - Deviance (showing properties that never appear in typical development);
  - Delayed behavior on finiteness (not deviant, but finiteness rates that are not significantly worse than younger controls who are matched on nonverbal MA and general language abilities);
  - Disrupted behavior on finiteness (not deviant, but finiteness rates that are significantly worse than younger nonverbal MA- and language-matched controls, cf. Rice, 2007).

Each of these four possibilities is a potential hypothesis. Of course, given the literature survey that we have just presented, we do not expect that children with ALI will show profile (3a), an intact pattern. So we can take profiles (3b, c, d) (deviant, delayed, disrupted) as hypotheses to test. We will not select one of these as our only hypothesis here; rather, the goal is to carry out a study that allows us to decide between these.

Although our study does not contain children with SLI, we can compare rates of finiteness with children with SLI from the literature. If our children with ALI show similar rates of finiteness as children with SLI functioning at comparable levels of cognitive and language abilities, this would support the idea that children with ALI have both ASD and SLI. Additionally, if children with ALI show a disrupted pattern of finiteness with respect to TD controls, then children with ALI would be like children with SLI with respect to this piece of language. However, if children with ALI show lower rates of finiteness than children with SLI, we can conclude that development of children with ALI is even more disrupted than that of children with SLI, and that there is more to language impairment in ALI than what is found in SLI. Moreover, children with ALI may potentially show a pattern of deviance that children with SLI do not show.

For children with ALN, grammatical deficits are not expected by definition. However, it is still important to compare knowledge of grammar in children with ALN relative to that of TD children to establish whether indeed children with ALN are “language normal” with respect to finiteness. It is always possible that a deficit in finiteness is not picked up by the standardized tests that establish a child as being ALN. We can thus consider the same hypotheses (3) for ALN.

Our choice of method of constrained elicited production, rather than natural production, is motivated by the following considerations. To determine the rate of finiteness, it is necessary to count not only children's production of relevant morphemes, but also omission thereof in obligatory contexts. Proportion of usage of an obligatory morpheme in obligatory contexts is the central measure that has been used in studies of production data concerning morphology since at least Brown (1973). These contexts can be difficult to determine precisely in natural production, especially in a language like English with a relatively impoverished system of verbal morphology. If a child omits verbal inflection, and also omits the subject, it is often impossible to tell whether this is a third person singular null subject with a bare stem (an optional infinitive, e.g., “[he] go”), or a first or second person null subject with the correct form of the verb with null inflection (e.g., “[I] go”). It is even more difficult to determine which tense was intended in a natural production context. These issues could be further compounded by the difficulties children with ASD have with attention and coherent dialogues, among other factors. Therefore, it is important to gather data on rates of finiteness in a controlled context, one in which the intended subject and the intended tense are unambiguously provided by the experimental context. The TEGI elicitation task does exactly this, providing past and present tense contexts with third person singular subjects, allowing for accurate measurement of finiteness rates. Furthermore, since TEGI was used to study language in other impairments (especially SLI), we have the possibility of comparing rates of finiteness in our participants with those in the literature.

As observed in the literature review, one of the challenges in making sense of the data in ASD is the heterogeneity in the verbal and nonverbal abilities in this population. To control for the heterogeneity of our participants' abilities, we follow recent literature and divide our participants into two groups based on their language-related phenotypes: Autism Language Normal (ALN) and Autism Language Impaired (ALI) (e.g., Kjelgaard and Tager-Flusberg, 2001; Roberts et al., 2004; Whitehouse et al., 2008; Perovic et al., 2013b). This yielded two relatively homogeneous ASD groups with respect to their productive and receptive language abilities, as well as nonverbal reasoning abilities.

We focus on comparisons of ALI and ALN groups and their matched TD control groups on finite responses on past and present tenses, as well as a recalculation based on all response types (percent correct vs. bare form vs. other responses), in obligatory contexts. We analyze participants' responses for other morphosyntactic aspects of the OI stage. We further evaluate our participants' finite responses via the criterion scores developed by Rice and Wexler (2001) to determine whether a participant scores at or below his or her chronological age.

To establish the influences of general grammar and vocabulary skills, nonverbal IQ and autistic symptomatology, we calculate correlations between children's finiteness levels and their scores on standardized tests of language, nonverbal reasoning, and measures that are used as a gold standard for diagnosis of ASD in the research literature, ADOS and ADI-R (Autism Diagnostic Observation Schedule: Lord et al., 2000; Autism Diagnostic Interview-Revised: Rutter et al., 2003). To our knowledge, this is the first such range of analyses described in the literature specifically for markers of tense. Our goal here is to understand whether deficits with finiteness correlate with autistic symptomatology, or whether they are independent of any autistic traits, and what the results mean for the computational linguistic abilities of these two groups of children with ASD. Finally, we compare our results with those from children with SLI from Rice and Wexler studies, to try to determine whether linguistic deficits in ASD are the same as linguistic deficits in SLI, with respect to early morphosyntax.

## Methods

### Participants

One hundred and sixty-four children participated in the study: 83 children with ASD (Chronological Age (CA): 4.35–16.3 years; 11 female)^2^, and 81 TD controls (CA: 3.5–17.1; 36 female). Their age and scores on standardized measures of nonverbal and verbal abilities are given in Table 1: NVIQ: the Matrices subtest of Kaufman Brief Intelligence Test (KBIT; Kaufman and Kaufman, 1990); expressive vocabulary: the Vocabulary subtest of KBIT (only for children with ASD); receptive vocabulary: the Peabody Picture Vocabulary Test Third Edition (PPVT-3; Dunn and Dunn, 1997); receptive grammar: Test of Reception of Grammar Second Edition (TROG-2; Bishop, 2003b).

**Table 1:** **Ages and mean scores (standard deviation) and ranges on standardized tests of language and cognition for the four participant groups**.

**Group**	**Age in years**	**KBIT matrices RS**	**KBIT matrices SS**	**KBIT vocabulary SS**	**PPVT-3 RS**	**PPVT-3 SS**	**TROG-2 RS**	**TROG-2 SS**
**ALI**	10.62 (3.07)	18.42 (8.82)	74.75 (22.90)	71.53 (18.72)	81.32 (30.21)	67.60 (16.55)	4.81 (3.69)	60.05 (7.96)
*N* = 37	6.42–16.32	0–39	40–112	40–107	29–147	40–100	0–12	55–85
**ALI-TD**	6.03 (2.63)	18.53 (8.39)	108.41 (11.66)		88.53 (35.8)	110.14 (11.64)	8.91 (5.41)	104.77 (14.95)
*N* = 36	3.5–13.2	4–39	88–143		34–177	84–135	0–19	83–139
**ALN**	9.52 (3.35)	27.13 (8.74)	108.13 (17.77)	108.82 (15.15)	124.11 (40.03)	106.98 (15.81)	13.41 (4.87)	97.39 (12.02)
*N* = 46	4.35–16.25	7–43	65–151	76–145	43–192	72–133	2–20	81–132
**ALN-TD**	9.54 (3.87)	26.91 (9.59)	108.89 (13.19)		125.56 (41.49)	110.27 (15.96)	14.59 (4.61)	104.23 (12.80)
*N* = 45	3.95–17.11	9–44	85–142		46–188	80–147	2–20	81–137
**Group differences**^a^	ALI-TD < ALI^***^, ALN^***^, ALN-TD^***^	ALI < ALN^***^, ALN-TD^***^. ALI-TD < ALN^**^, ALN-TD^**^	ALI < ALI-TD^***^, ALN^***^, ALN-TD^***^	ALI < ALN^***^	ALI < ALN^***^, ALN-TD^***^. ALI-TD < ALN^**^, ALN-TD^**^	ALI < ALI-TD^***^, ALN^***^, ALN-TD^***^	ALI < ALI-TD^***^, ALN^***^, ALN-TD^***^. ALI-TD < ALN^*^, ALN-TD^***^	ALI < ALI-TD^***^, ALN^***^, ALN-TD^***^. ALN < ALI-TD^*^, ALN-TD^*^

* *p < 0.05*,

** *p < 0.01*,

*** *p < 0.001*.

*Measures on which relevant groups were individually matched: KBIT Matrices raw score for ALI and ALI-TD, and ALN and ALN-TD. ALI, Autism Language Impaired; ALN, Autism Language Normal; TD, Typically Developing controls; RS, Raw Score; SS, Standard Score*.

a *Significances for group differences are based on pairwise comparisons (Bonferroni corrected) following a MANOVA with age, raw and standard scores as dependent variables, the four participant groups as the independent variable, and gender as a covariate. There was not a significant effect of gender F_(7, 144)_ = 1.142, p = n.s., but a significant effect of group F_(21, 414)_ = 14.83, p < 0.001; Wilks' lambda = 0.2*.

This study was approved by the Committee on the Use of Humans as Experimental Subjects at the Massachusetts Institute of Technology. Written parental consent was obtained for all participants.

Fifty-eight participants with ASD were recruited with the help of the Division of Developmental Medicine, Boston Children's Hospital (BCH), Harvard Medical School, for the Simons Simplex Collection of the phenotypic and genetic factors in ASD (Lord et al., 2012). Twenty-five were recruited via parent support groups based in the greater Boston, Massachusetts area. All participants met the clinical criteria for ASD according to *DSM-IV* (American Psychiatric Association, 2000), which were confirmed for 49 participants by BCH via Autism Diagnostic Interview–Revised and Autism Diagnostic Observation Schedule, scores for which were provided to us^3^. In six of these participants, diagnosis was confirmed by either ADI-R or ADOS, but not both^4^.

In part following Perovic et al. (2013b) we divide our participants into two groups based on their language abilities: Autism Language Normal (ALN, *n* = 46, 5 female) and Autism Language Impaired (ALI, *n* = 37, 6 female), according to their scores on the Vocabulary subtest of KBIT, a measure of expressive vocabulary, and measures of receptive vocabulary, PPVT-3, and receptive grammar, TROG-2 (cf. Whitehouse et al., 2008). To balance the one measure of grammar against the two measures of vocabulary, we used the procedure in (4) and (5) to form groups^5^.

(4) We classified children as ALN if they scored at or above the 10th percentile (i.e., a standard score (SS) of 81 or above) in TROG-2 and in at least one of the two vocabulary tests (KBIT-Vocabulary and PPVT-3). 96% of ALN participants were at or above the 10th percentile on all three tests.
(5) Children were classified as ALI if their score was below the 10th percentile in TROG-2, and below the 10th percentile in at least one of the two vocabulary tests. This was true for 84% of ALI participants. Five participants who had good scores on both vocabulary tests, but with TROG-2 scores in the impaired range (SS of 69 or below), were also assigned to the ALI group. Finally, one participant who scored below the 10th percentile on both vocabulary tests, but had SS of 85 on TROG-2, joined the ALI group^6^.

TD controls were recruited from Boston and Cambridge, Massachusetts area daycares and afterschool programs, and had no known cognitive or language delays or hearing impairments. They were individually matched to ASD participants on the raw score of KBIT Matrices, forming two groups: TD controls of ALI group (ALI-TD, *n* = 36, 14 female) and TD controls of ALN group (ALN-TD, *n* = 45, 22 female)^7^.

Table 1 shows that the ALN and ALI groups were also matched to their respective control groups on verbal MA (raw scores on PPVT-3), while the ALN group was also matched to their TD control group on receptive grammar (raw scores on TROG-2). It should be noted that the ALI group's raw scores on receptive grammar were age-equivalent to 4;5 in TD, which is much lower than the ALI-TD group's chronological age of 6;0. The ALN and ALI groups were matched on age, but on all other measures the ALN group had significantly higher scores than the ALI group. Finally, the ALN-TD and ALI groups are comparable in their age.

The groups were not gender-matched due to a limited sample size of TD controls. To control for effects of gender, we included this variable as a covariate in our subsequent analyses: no significant effect of gender was found.

Finally, because not all children with ASD had ADOS and ADI-R scores, we did not match the ALI and ALN groups on these measures. It is notable that for the subgroups of ALN (*n* = 28) and ALI (*n* = 21) that had these scores, there were significant group differences on ADOS Communication [ALI mean = 4.52, *SD* = 2.04; ALN mean = 2.04, *SD* = 1.64; *t*_(47)_ = 4.73, *p* < 0.001] and ADOS Social interaction [ALI mean = 8.86, *SD* = 3.34; ALN mean = 5.25, *SD* = 1.43; *t*_(47)_ = 5.14, *p* < 0.001]. The higher scores here indicate a greater severity of ASD symptoms. There were no other significant differences (after correcting for multiple comparisons) between ASD groups on other ADOS or any of the ADI-R scores.

### Experimental materials and procedure

Three picture probes of the Test of Early Grammatical Impairment (TEGI, Rice and Wexler, 2001) were used: phonological probe, present tense, and past tense.

The phonological probe determined whether children could pronounce the consonant sounds relevant to present and past tense inflections, /*s*/, /*z*/, /*t*/, and /*d*/. Participants had to correctly articulate at least four of five words in order to pass. If participants could not provide the required word on their own, they were asked to repeat it after the experimenter.

The present tense probe assessed whether children could produce third person singular inflection using a representative picture for a profession and the following prompt: “This is a teacher. Tell me what a teacher does.” If participants replied with a plural subject, e.g., “Teachers teach” or without a subject, e.g., “Teach,” they were reprompted to provide an answer with a singular subject. Following the manual, we used such phrases as “Say a whole sentence,” or “Start with he or she.” If that did not work, the experimenter started the sentence for the participant, saying, e.g., “A teacher…” after which the participant sometimes completed the sentence. Finally, if a child produced an answer which was semantically appropriate but was neither finite nor nonfinite, e.g., a progressive form, the experimenter agreed with the participant, and then prompted him or her with, “Tell me what else a teacher does?” Often, especially with lower functioning or younger participants, these prompts did not produce the desired kind of response, and we simply recorded whatever answers the participants provided. There was one training example and 10 trials.

The past tense probe assessed whether children could produce the -*ed* suffix on regular verbs, or the irregular past tense form of irregular verbs. The prompt involved two pictures, with one picture showing ongoing action, e.g., “Here the girl is skating.” The next picture showed the action completed and was accompanied with the prompt “Here she is done,” followed by “Tell me what she did.” The same reprompting questions were used by the experimenters in this probe as for the present tense probe, above. Occasionally, children produced a regular verb instead of an irregular verb, and re-prompting did not yield the correct verb. There were two training examples, and 10 trials for regular verbs and 8 for irregular verbs.

### Scoring and analysis

Answers were scored following the instructions in the TEGI Manual (Rice and Wexler, 2001). Correct (finite) answers included appropriate *-s* or *-ed* inflections on the verbs or the correct irregular past tense form. Incorrect (nonfinite) answers included bare stems of the verbs. Over-regularized verb forms, e.g., “digged,” were scored as correct, i.e., finite. The rate of finiteness was calculated as: finite responses/(finite + nonfinite responses). The rate of over-regularization was analyzed as the number of over-regularized forms out of the total number of irregular verbs that were scorable, including nonfinite (bare) forms. A series of univariate ANOVAs was performed for rates of finiteness, following analyses carried out in Rice and Wexler studies. Group differences throughout were identified by pairwise comparisons, Bonferroni corrected by SPSS Version 23.

“Unscorable” answers, ones that we did not purposefully elicit, were those without verbs (nouns only), inappropriate tenses for the prompt, and responses with “does,” “did” or “done.” Repeated use of “he/she finished” was also marked unscorable (c.f. Rice and Wexler, 2001). Verbs with the *-ing* suffix, whether or not produced with a relevant auxiliary, were also marked as unscorable, following Rice and Wexler (2001). Unscorable answers were not included in the denominator for percent of finite responses.

The distribution by all response types, that is percentage of correct forms vs. bare forms vs. unscorable or unattempted answers, was also analyzed separately, following Roberts et al. (2004). Here, the number of both attempted and unattempted verbs was included in the denominator. In this case, a series of univariate ANOVAs was performed for each tense and response type.

Unscorable answers were analyzed separately for those participants that produced them for any tense or other errors. All answers were analyzed for other aspects of morphosyntax, namely subject-verb agreement and case.

We also counted whether there was an overt or a null subject with an inflected or nonfinite (bare) verb. Because the only appropriate answers to our elicitation task contain third person singular subjects and verbs (and also because when the children *do* produce a subject, it and the verb are overwhelmingly in third person, if the verb is finite), it is highly unlikely that participants intended first or second person subjects as their null subjects, so we count bare stem verbs without subjects as being nonfinite. Responses in which subjects were prompted by the experimenter were excluded from these counts. Only responses in which it was clear that the participants produced or omitted a subject by themselves (without an extra prompt) were counted. Correct responses consisted of an overt third person singular nominative pronoun subject (or a noun phrase) and a finite verb. The relationship between presence of subjects and finiteness of verbs was tested using chi-square.

## Results

A few participants from the ALI group did not produce any scorable responses for certain tenses, and were thus excluded from analyses for that tense: two were excluded from analyses for present tense (*n* = 34), one from regular past tense (*n* = 35), and three from irregular past tense (*n* = 33). Their scorable responses for the remaining tenses were included, so that for all past tense there were 36 ALI participants.

For one third of ALI-TD and about half of ALN-TD participants, detailed scores of the probes were not available, just the rates of finiteness for present and all past tenses in percent (included in Table 2 and counted for Criterion scores in **Table 14**). There were no significant differences in the rates of finiteness for present and overall past tenses between the TD subgroups with vs. without such scores. For this reason, we move forward with the reduced number of TD participants with respect to the details of regular and irregular past tenses (Table 3), and reanalysis by response type (Tables 4–**6**) (*n* = 26 for ALI-TD, *n* = 24 for ALN-TD)^8^.

**Table 2:** **Percent finite responses with mean (standard deviation) for present tense and all past tense (regular and irregular combined) probes**.

**Group**	**Rate of finiteness for present tense**	**Rate of finiteness for all past tenses**	**Intra group differences**
**ALI**	65.30 (35.67)	67.83 (33.6)	n.s.
**ALI-TD**	90.53 (21.64)	92.43 (18.41)	n.s.
**ALN**	87.80 (26.06)	92.82 (18.39)	n.s.
**ALN-TD**	98.76 (6.15)	96.92 (12.10)	n.s.
**Inter group differences**	ALI < ALI-TD^***^, ALN^***^, ALN-TD^***^	ALI < ALI-TD^***^, ALN^***^, ALN-TD^***^	

*** *p < 0.001*.

**Table 3:** **Percent finite responses with mean (standard deviation) for regular and irregular past tense probe**.

**Group**	**The rate of past regular finite form**	**The rate of past irregular finite form (sum of correct and over-regularized forms)**	**The rate of past irregular correct form**	**The rate of past over-regularized form**	**Intra group differences between regular and irregular finite responses**
**ALI**	70.85 (35.61)	72.54 (30.90)	47.54 (32.22)	25.00 (28.69)	n.s.
**ALI-TD**	95.80 (10.25)	91.19 (17.41)	61.97 (29.50)	29.21 (21.79)	n.s.
**ALN**	92.79 (20.40)	92.19 (19.35)	71.50 (32.82)	20.69 (24.54)	n.s.
**ALN-TD**	93.96 (17.13)	95.95 (15.78)	82.87 (26.16)	13.08 (18.90)	n.s.
**Inter group differences**	ALI < ALI-TD^**^, ALN^***^, ALN-TD^**^	ALI < ALI-TD^*^, ALN^**^, ALN-TD^*^	ALI < ALN^**^, ALN-TD^**^	n.s.	

* *p < 0.05*,

** *p < 0.01*,

*** *p < 0.001*.

**Table 4:** **Percent of response types with mean (standard deviation), and sums of responses, for present tense probe**.

**Group (total responses)**	**Correct**	**Bare stem**	**Not scorable**	**Not attempted**
**ALI**, (363)	53.10 (38.91), 193	19.68 (21.93), 72	22.22 (25.98), 80	5.00 (19.78), 18
**ALI-TD**, (260)	90.39 (19.90), 235	7.31 (15.64), 19	2.31 (9.92), 6	0 (0), 0
**ALN**, (451)	85.94 (26.80), 385	10.75 (21.66), 49	3.31 (6.38), 17	0 (0), 0
**ALN-TD**, (240)	97.08 (9.99), 233	1.67 (8.17), 4	1.25 (6.12), 3	0 (0), 0
**Group differences**	ALI < ALI-TD^***^, ALN^***^, ALN-TD^***^	ALI > ALN-TD^*^	ALI > ALI-TD^***^, ALN^***^, ALN-TD^***^	n.s.

* *p < 0.05*,

*** *p < 0.001*.

### Phonological probe

All ALN and ALN-TD participants passed all 4 subtests of the phonological probe. One ALI participant did not pass one of the four subtests of the probe, the /*z*/ sound, but passed the other sound relevant to present tense: /*s*/. He scored 43% on present tense, which is identical to his performance on past tense (43%). One child in the ALI-TD group did not pass the /*t*/ sound. This child scored 100% correct on past tense, however.

### Rates of finiteness

#### Present and past tenses

The participants' mean rates of finiteness, that is the mean of individual finite responses divided by the individual's sum of finite and nonfinite (bare) responses, are illustrated in Table 2 for present tense and all past tense (average of finite irregular and regular past tense responses). A series of univariate ANOVAs was performed, with participant group (ALI, ALI-TD, ALN, ALN-TD) as between subjects factor and percent of finite responses for each tense as the dependent measure. Gender was entered as a covariate. While there was no significant effect of gender for any tense, the effect of group was significant for present tense *F*_(3, 156)_ = 11.37, *p* < 0.001 ($\eta_{p}^{2}$ = 0.18) and for all past tense *F*_(3, 158)_ = 13.23, *p* < 0.001 ($\eta_{p}^{2}$ = 0.2). Pairwise comparisons (Bonferroni corrected) indicated that the ALI group performed well below all other groups (all *ps* < 0.001) on both tenses, while the ALN group performed no differently from either of the TD control groups on the same probes. Differences between present and all past tense performance were not significant in each group.

#### Regular and irregular past tenses

Table 3 shows the specifics of participants' finiteness rates for regular and irregular past tense verbs. The finiteness rate for regular past tense was calculated as in the previous section for present and all-past tenses. The rate of morphologically correct irregular past tense forms was derived by dividing the number of such forms by the total number of scorable irregular verbs (including bare forms) for each participant. The rate of over-regularization was similarly calculated as the number of over-regularized forms divided by the total number of scorable irregular verbs. The rate of finite responses for irregular past tense was the sum of the rate of correct irregular form responses and the rate of over-regularized form responses. The statistical analyses were performed as in the previous section.

We find a significant effect of group for regular past tense finite responses *F*_(3, 125)_ = 7.63, *p* < 0.001 ($\eta_{p}^{2}$ = 0.16), irregular finite responses *F*_(3, 123)_ = 6.36, *p* < 0.001 ($\eta_{p}^{2}$ = 0.13), and irregular correct form responses *F*_(3, 123)_ = 6.18, *p* = 0.001 ($\eta_{p}^{2}$ = 0.13), but not for over-regularized past tense forms. Gender was not significant as a covariate. Pairwise comparisons (Bonferroni corrected) indicated that the ALI group performed well below all other groups (*p* = 0.001 for ALI-TD, *p* < 0.001 for ALN, and *p* = 0.009 for ALN-TD) on regular past tense. On finite responses for irregular past tense, the ALI group was also worse than other groups: ALI-TD (*p* = 0.025), ALN (*p* = 0.001), and ALN-TD (*p* = 0.011). On the correct forms for irregular past tense, however, the ALI group did not differ from the ALI-TD group, but did perform worse than the ALN and the ALN-TD groups (*p* = 0.005 and *p* = 0.002, respectively). The ALN group did not differ from either of the TD control groups. No significant differences were observed within groups between regular and irregular finite tense responses.

### Performance by response type

We reanalyzed our data by percent of all response types, so that the denominator includes all verbs in the probe, not just those responses that are scorable. Sums of raw numbers are also indicated in Tables 4–**6**. A series of univariate ANOVAs was performed for each tense and response type, with participant group (ALI, ALI-TD, ALN, ALN-TD) as the between-subjects factor. Gender was added as a covariate.

There was significant effect of group for most response types in most tenses. There was no significant effect of gender. Pairwise comparisons (Bonferroni corrected) indicated that the ALI group performed worse than other groups on percent correct, and had more percent bare, percent unscorable and percent unattempted (no response) responses.

For present tense (Table 4), the following variables had a significant effect of group: correct *F*_(3, 126)_ = 14.17, *p* < 0.001 ($\eta_{p}^{2}$ = 0.25, ALI < all others, all *ps* < 0.001), bare stem *F*_(3, 126)_ = 3.87, *p* = 0.011 ($\eta_{p}^{2}$ = 0.08, ALI > ALN-TD *p* = 0.011 only), and unscorable *F*_(3, 126)_ = 13.65, *p* < 0.001 ($\eta_{p}^{2}$ = 0.25, ALI > all others, all *ps* < 0.001). Unattempted responses were not significantly different among groups *F*_(3, 126)_ = 1.85, *p* = n.s.

For regular past tense (Table 5), the following variables had significant effect of group: correct *F*_(3, 126)_ = 15.77, *p* < 0.001 ($\eta_{p}^{2}$ = 0.27, ALI < all others, all *ps* < 0.001), unscorable *F*_(3, 126)_ = 11.7, *p* < 0.001 ($\eta_{p}^{2}$ = 0.22, ALI > ALN and ALN-TD, *p* < 0.001, and ALI > ALI-TD, *p* = 0.001), and unattempted *F*_(3, 126)_ = 4.82, *p* = 0.003 [$\eta_{p}^{2}$ = 0.10, ALI > ALI-TD (*p* = 0.038) and ALN (*p* = 0.004)]. Bare stem responses were not significantly different among groups *F*_(3, 126)_ = 2.25, *p* = n.s.

**Table 5:** **Percent of response types with mean (standard deviation), and sums of responses, for regular past tense probe**.

**Group (total responses)**	**Correct**	**Bare stem**	**Not scorable**	**Not attempted**
**ALI**, (363)	57.39 (37.64), 208	15.06 (17.55), 55	16.74 (22.12), 61	10.81 (26.55), 39
**ALI-TD**, (261)	92.34 (16.32), 241	4.20 (10.25), 11	3.46 (13.84), 9	0 (0), 0
**ALN**, (452)	92.57 (20.37), 418	7.21 (20.40), 33	0.22 (1.49), 1	0 (0), 0
**ALN-TD**, (240)	92.92 (18.05), 223	5.83 (16.92), 14	1.25 (4.48), 3	0 (0), 0
**Group differences**	ALI < ALI-TD^***^, ALN^***^, ALN-TD^***^	n.s.	ALI > ALI-TD^**^, ALN^***^, ALN-TD^***^	ALI > ALI-TD^*^, ALN^**^

* *p < 0.05*,

** *p < 0.01*,

*** *p < 0.001*.

For irregular past tense (Table 6), the following variables had significant effect of group: finite forms *F*_(3, 126)_ = 15.75, *p* < 0.001 ($\eta_{p}^{2}$ = 0.27, ALI < all others, all *ps* < 0.001), correct irregular form *F*_(3, 126)_ = 10.91, *p* < 0.001 [$\eta_{p}^{2}$ = 0.21, ALI < ALI-TD (*p* = 0.039), ALI < ALN and ALN-TD (*p* < 0.001)], unscorable *F*_(3, 126)_ = 8.89, *p* < 0.001 [$\eta_{p}^{2}$ = 0.18, ALI >ALI-TD (*p* = 0.004), ALI > ALN (*p* < 0.001), ALI > ALN-TD (*p* = 0.002)], and unattempted *F*_(3, 126)_ = 4.56, *p* = 0.005 [$\eta_{p}^{2}$ = 0.1, ALI >ALI-TD (*p* = 0.042), ALI > ALN (*p* = 0.005)]. For bare stem and over-regularized form responses, the effect of group was not significant.

**Table 6:** **Percent of response types with mean (standard deviation), and sums of responses, for irregular past tense probe**.

**Group (total responses)**	**Finite (sum of correct and over-regularized forms)**	**Correct form**	**Over-regularized form**	**Bare stem**	**Not scorable**	**Not attempted**
**ALI**, (289)	55.18 (38.28), 161	37.34 (32.68), 109	17.84 (23.84), 52	14.07 (16.60), 40	18.25 (26.90), 52	12.5 (31.05), 36
**ALI-TD**, (207)	89.42 (20.52), 185	61.33 (29.58), 127	28.09 (22.00), 58	7.21 (13.31), 15	3.37 (13.02), 7	0 (0), 0
**ALN**, (358)	91.11 (20.61), 327	70.98 (33.08), 255	20.13 (24.15), 72	6.87 (16.39), 24	1.75 (5.44), 6	0.28 (1.86), 1
**ALN-TD**, (193)	94.91 (16.31), 183	82.18 (26.82), 158	12.73 (18.59), 25	4.05 (15.78), 8	1.04 (5.10), 2	0 (0), 0
**Group differences**	ALI < ALI-TD^***^, ALN^***^, ALN-TD^***^	ALI < ALI-TD^*^, ALN^***^, ALN-TD^***^	n.s.	n.s.	ALI > ALI-TD^**^, ALN^***^, ALN-TD^**^	ALI > ALI-TD^*^, ALN^**^

* *p < 0.05*,

** *p < 0.01*,

*** *p < 0.001*.

### Unscorable responses: tense substitutions, use of progressives, and auxiliary omissions

In the ALI group, there was a total of 193 “unscorable” answers for 26 participants, a rate of 19.0% given 1,015 total responses across tenses and participants.

The ALN group had just 24 unscorable responses in 16 participants, a rate of 1.9% given 1,261 total responses across tenses and participants.

Table 7 summarizes participants' unscorable responses for present and past tense probes, and also the correct and bare stem responses for comparison. The total number of unscorable responses characterized in the table for the ALI group is greater than 193 because some participants' responses included multiple types of answers.

**Table 7:** **Number of each type of response, including correct, nonfinite, and “unscorable” responses (with number of participants giving each type of response)**.

	**ALI**		**ALN**	
	**Present tense probe**	**All past tense probe**	**Present tense probe**	**All past tense probe**
Simple past (finite)	2 (2)	369 (35)	0	745 (44)
Simple present (finite)	193 (29)	8 (4)	385 (43)	0
Bare stem (nonfinite)	72 (22)	95 (25)	49 (13)	57 (9)
Present progressive (present tense auxiliary + progressive participle)	24 (11)	21 (8)	3 (3)	0
Past progressive	0	1	0	0
Progressive participle **without** auxiliary	21 (9)	35 (9)	3 (2)	2 (2)
Present tense auxiliary with bare verb (omission of *-ing*)	2 (2)	1	0	0
Copula “is”	5 (2)	0	0	0
Future “is going to”	2 (1)	1	0	0
“s/he is (all) done”	0	10	0	2 (2)
“Does”	3 (3)	0	2 (2)	0
Modal	1	0	0	0
“Finished”	0	13 (4)	0	0
“Did”	0	8 (5)	0	0
Nouns for subject	7 (3)	2 (2)	0	0
Nouns for object	19 (7)	6 (3)	1	0
Preposition phrases	7 (6)	0	0	0
Adjectives	3 (2)	0	0	0
No response	18 (3)	75 (7)	0	1
Unscorable due to experimenter issues e.g., responses were unintelligible or there was disagreement between scorers	17 (10)		11 (9)	

#### Unscorable tense responses in the ALI group

Overall, the ALI participants were hardly ever confused between the simple past and simple present tenses, giving a total of 10 errors out of 572 uses of simple tenses for all participants for all probes, a rate of 1.7%.

In the present tense probe, the progressive participle -*ing* was used 45 times, 12.4% of the 363 total responses for that probe. Of these 45, 24 were with present tense auxiliary, and 21 were without auxiliary, a 46.7% rate of auxiliary omission.

In the past tense probe, present tenses were used 40 times, 6% of the 652 total responses for that probe. The majority of these (21) were in present progressive tense, with 2 participants contributing 14 of these. These two participants produced proper past tense morphology only four times between them. Other present tense responses included simple present, present tense auxiliary with bare verb, and “s/he is (all) done.”

A present participle (stem + *ing*) without an auxiliary occurred an additional 35 times (5.4% of total responses for past tense probe), with two participants contributing 24 of those (these are different participants from the 14 present progressive responses, above). This yields a 62.5% auxiliary omission rate. Participants who omitted auxiliaries were not significantly more likely to produce bare stem verbs.

In the past tense probe, there was one case of simple present tense together with past tense overregularization, “catchesed.” Finally, there was one future tense that is interpreted as future/intention, “she's gonna run” for the picture with a girl tying her shoelaces.

Across the probes, there were three instances (one each from three ALI participants) using the auxiliary “is” and a bare form of a verb, omitting -*ing*: in present tense, “a girl is ride(ing) her bike,” “he's take,” giving a rate of 8% of -*ing* omission for finite progressive tense responses; in the past tense, “the boy is splash” (5%) ^9^. There was also “he's clean” in the past tense probe for a picture of a boy having brushed his hair, and this could be either missing -*ing* or adjective use.

#### Unscorable tense responses in the ALN group

Nobody in the ALN group misused past tense in the present tense probe and vice versa, that is 0 out of 1,130 simple tense responses for all ALN participants in all probes. In addition to those responses detailed in Table 7, there was also one instance of negation use in the present without auxiliary, “baby not get hurt.” Rate of -*ing* omission was 0%. Rate of auxiliary omission in present progressive use was 62.5% (5/8) responses across all probes. Participants who omitted auxiliaries were not significantly more likely to produce bare stem verbs.

### Case and subject-verb agreement with respect to number and person

The responses of participants with ALI and ALN were also examined to establish the presence of any difficulties with morphosyntax, specifically with case marking and subject-verb agreement. We found no such errors: for example, no participant used a first or second person pronoun with third person singular verbal inflection in present tense, and none misused case on pronouns.

There were only three instances of first person pronoun in nominative case for the subject, and only two in accusative case for the object. The second person pronoun “you” was used for a subject by only one person in two complex sentences. “You” was regularly used for objects, especially with a picture of a dad or a nurse, “…helps you,” a total of 11 times for 8 ASD participants. The determiner “your” was used primarily with a picture of a dentist, e.g., “(…) clean(s) your teeth,” a total of 19 times for 18 ALI/ALN participants.

All pronouns that were used were in appropriate cases for their sentence role, with nominative for subjects, accusative for objects, possessive/genitive in relevant constructions.

For third person singular present tense, pronouns “he” or “she” or “it” were 100% correctly used, as the subject by 17 ALI participants: 49 times with finite verbs and 10 times with nonfinite verbs; and by 11 ALN participants: 46 times with finite verbs and 3 times with nonfinite verbs. Noun phrases were used as the subject by 10 ALN participants: 56 times for finite and 3 times for nonfinite verbs; and by 9 ALI participants: 33 times for finite verbs and 8 times for nonfinite verbs. There were no instances of incorrect use.

Because the probes focused on the elicitation of singular subjects, plural subjects were not purposefully elicited. Children with ASD did not make any agreement errors here, with the two overt plural subjects that two participants in the ALN group produced showing correct agreement. One plural subject in the ALI group was also appropriate.

### Null vs. overt subjects with nonfinite and finite verbs

The presence of null or overt subjects was calculated in 70% of ALI participants (*n* = 26) and 41% of ALN participants (*n* = 19)^10^.

We begin by comparing null vs. overt subjects within and between groups (collapsing across non/finite verbs) (Table 8). There were no significant differences between groups for these measures: both groups produced similar overall proportions of null and overt subjects. Within groups, for both ALI and ALN, the difference between the rate of null and overt subjects in past tense was significant: *t*_(23)_ = 2.98, *p* = 0.007 for the ALI group, and *t*_(16)_ = 3.05, *p* = 0.008 for the ALN group. This difference between past and present tenses is likely due to the different probes. The present tense probe asked e.g., what “a nurse” generically does, and it seems quite felicitous to respond without a subject, whereas the past tense probe asked which specific completed activity a specific person, e.g., “the girl,” carried out, and a null subject seems to be not nearly as felicitous.

**Table 8:** **Proportions for null and overt subjects in ASD across verb forms**.

	**Present tense**			**Past tense**		
	**Overt subjects**	**Null subjects**	**Intra group differences**	**Overt subjects**	**Null subjects**	**Intra group differences**
**ALI**	53.44% (101/189)	46.56% (88/189)	n.s.	73.49% (219/298)	26.51% (79/298)	^**^
**ALN**	64.67% (108/167)	35.33% (59/167)	n.s.	78.25% (223/285)	21.75% (62/285)	^**^
**Group differences**	n.s.	n.s.		n.s.	n.s.	

** *p < 0.01*.

Table 9 indicates the relevant sums across participants and the rates of null subjects for each verb type. For present tense, there was a significant difference for correct responses (overt subject with finite verb) between the ALI and ALN groups, 43.91% (83/189) vs. 61.08% (102/167) respectively, *F*_(1, 36)_ = 5.4, *p* = 0.026 ($\eta_{p}^{2}$ = 0.13). For past tense, there were two significant differences between the groups. The ALN group gave more correct responses than the ALI group, 77.54% (221/285) vs. 59.40% (177/298), respectively, *F*_(1, 36)_ = 11.36, *p* = 0.002 ($\eta_{p}^{2}$ = 0.24). The ALI group produced significantly more overt subjects with nonfinite verbs than the ALN group, 14.09% (42/298) vs. 0.70% (2/285), respectively, *F*_(1, 36)_ = 6.96, *p* = 0.012 ($\eta_{p}^{2}$ = 0.16). The rate of null subjects produced with either verb form in the past or present tense did not differ between the ALN and ALI groups.

**Table 9:** **Counts and rates of null subjects for finite and nonfinite verbs in ASD, and likelihood ratios (of having a null subject with a nonfinite verb compared to having a null subject with a finite verb)**.

	**Present**		**Past**	
	**Finite verb**	**Nonfinite verb**	**Finite verb**	**Nonfinite verb**
**ALI**				
**Overt subject**	83	18	177	42
**Null subject**	50	38	47	32
**Rate of null subjects for each verb type**	37.59%	67.86%	20.98%	43.24%
Ratio	1.8		2.1	
**ALN**				
**Overt subject**	102	6	221	2
**Null subject**	43	16	35	27
**Rate of null subjects for each verb type**	29.66%	72.73%	13.67%	93.10%
Ratio	2.5		6.8	

Chi-square tests for independence were used to examine the relationship between null/overt subjects and non/finite verbs within each group. We find significant relationships in the ALI group for present tense, ${\chi^{2}}_{\left(1,N=189\right)}=14.51,$ *p* < 0.0001, and for past tense ${\chi^{2}}_{\left(1,N=298\right)}=14.15,$ *p* = 0.0001. Similar significances were observed for the ALN group for present tense, ${\chi^{2}}_{\left(1,N=167\right)}=15.51,$ *p* < 0.0001, and for past tense ${\chi^{2}}_{\left(1,\;N=285\right)}=96.55,$ *p* < 0.0001. Thus, there is a higher rate of null subjects with nonfinite verbs, and a higher rate of overt subjects for finite verbs, for both ALN and ALI groups and both tenses. The trends are consistent with what was previously reported for elicited production in controlled contexts for very young TD children (Schütze and Wexler, 2000). Table 9 also presents likelihood ratios of having a null subject with nonfinite verb compared to having a null subject with a finite verb, which are greater than 1 in all cases.

### Correlations between tense marking and chronological age and standardized tests

Pearson Bivariate Correlations^11^ for ALN and ALI groups were calculated between response variables indicated in Tables 2 and 3, as well as composite tense (mean of finite responses of present, past regular and past irregular tenses) and CA, and SS on NVIQ (KBIT Matrices), KBIT expressive Vocabulary, receptive vocabulary (PPVT-3) and receptive grammar (TROG-2).

In the case of the ALI group, we find significant correlations for different aspects of tense with receptive and productive vocabulary, receptive grammar, as well as NVIQ, but not with CA (Table 10).

**Table 10:** **Correlations for the ALI group between tense marking performance and age and standard scores on KBIT matrices and vocabulary, PPVT-3 and TROG-2**.

	**Chronological age**	**KBIT matrices**	**KBIT vocabulary**	**PPVT-3**	**TROG-2**
**Present finite form%**	0.329	0.179	0.322	0.538^**^	0.399^*^
**Past regular finite form%**	0.152	0.265	0.352^*^	0.555^**^	0.352^*^
**Past irregular correct form%**	0.293	0.243	0.370^*^	0.328	−0.005
**Past over-regularized form%**	−0.102	0.101	−0.145	0.14	0.316
**Past irregular finite forms%**	0.211	0.348^*^	0.251	0.472^**^	0.288
**All past finite forms%**	0.24	0.410^*^	0.464^**^	0.645^***^	0.388^*^
**Composite finite tense%**	0.293	0.339^*^	0.431^**^	0.618^***^	0.411^*^

* *p < 0.05*,

** *p < 0.01*,

*** *p < 0.001*.

In the ALN group, on the other hand, finiteness strongly correlates with CA only (Table 11).

**Table 11:** **Correlations for the ALN group between tense marking performance and age and standard scores on KBIT matrices and vocabulary, PPVT-3 and TROG-2**.

	**Chronological age**	**KBIT matrices**	**KBIT vocabulary**	**PPVT-3**	**TROG-2**
**Present finite form%**	0.420^**^	−0.037	−0.107	−0.004	−0.075
**Past regular finite form%**	0.376^*^	0.152	0.075	0.221	0.357^*^
**Past irregular correct form%**	0.735^***^	0.14	−0.063	0.279	0.041
**Past over-regularized form%**	−0.639^***^	−0.082	0.019	−0.269	−0.036
**Past irregular finite forms%**	0.436^**^	0.145	−0.13	0.132	0.024
**All past finite forms%**	0.424^**^	0.159	0.018	0.213	0.273
**Composite finite tense%**	0.455^**^	0.054	−0.06	0.092	0.075

* *p < 0.05*,

** *p < 0.01*,

*** *p < 0.001*.

### Correlations between tense marking and ASD diagnostic measures

In the ALI group, there were significant negative correlations between knowledge of finiteness, including composite tense, and ADI-R scores on the Current Algorithm on domains of Social Interaction, Verbal and Nonverbal Communication, and Behavior (Table 12). ADOS scores on the domains of Communication and Social Interaction correlated significantly with irregular finite past and regular finite past forms respectively (Table 13). ADOS Behavior/Interaction scores correlated with regular finite past tense form. Notably, no ADOS scores nor ADI-R Diagnostic Algorithm scores correlated significantly with composite tense.

**Table 12:** **Correlations between tense marking and ADI-R Current algorithm scores for the ALI group (with range of number of participants for each subtest)**.

	**ADI-R Current social interaction (14–17)**	**ADI-R Current verbal communication (14–17)**	**ADI-R Current nonverbal communication (10–13)**	**ADI-R Current behavior (14–17)**
**Present finite form%**	−0.737^**^	−0.796^***^	−0.862^**^	−0.597^*^
**Past regular finite form%**	−0.605^*^	−0.582^*^	−0.614^*^	−0.349
**Past irregular correct form%**	−0.231	−0.199	−0.337	−0.278
**Past over-regularized form%**	−0.590^*^	−0.551^*^	−0.433	−0.351
**Past irregular finite forms%**	−0.767^**^	−0.701^**^	−0.666^*^	−0.597^*^
**All past finite forms%**	−0.678^**^	−0.615^*^	−0.697^*^	−0.45
**Composite finite tense%**	−0.704^**^	−0.700^**^	−0.787^**^	−0.552^*^

* *p < 0.05*,

** *p < 0.01*,

*** *p < 0.001*.

**Table 13:** **Correlations between tense marking and ADOS measures for the ALI group (with range of number of participants for each subtest)**.

	**ADOS communication (17–21)**	**ADOS social interaction (17–21)**	**ADOS imagination/creativity (9–10)**	**ADOS behavior/interaction (17–21)**
**Present finite form%**	−0.269	−0.332	0.107	−0.144
**Past regular finite form%**	−0.314	−0.472^*^	−0.078	−0.466^*^
**Past irregular correct form%**	−0.32	−0.188	0.51	0.377
**Past over-regularized form%**	−0.237	−0.053	−0.499	−0.305
**Past irregular finite forms%**	−0.549^*^	−0.244	−0.082	0.116
**All past finite forms%**	−0.359	−0.434	−0.009	−0.335
**Composite finite tense%**	−0.291	−0.375	−0.056	−0.278

* *p < 0.05*.

For the ALN group, there were no significant correlations between ADOS and ADI-R scores and composite tense. Only two other measures of tenses had significant correlations. Correct form of irregular past tense [*r*_(25)_ = −0.412, *p* < 0.05] and levels of over-regularized responses [*r*_(25)_ = 0.423, *p* < 0.05] significantly correlated with ADOS Communication scores; correct form of irregular past tense also correlated with ADI-R Diagnostic Algorithm scores on Social Interaction [*r*_(25)_ = 0.385, *p* < 0.05].

### Tense marking performance according to the TEGI criterion scores

While the ALI group is clearly impaired on finiteness, the ALN group was not significantly worse than its controls. Analysis of the TEGI criterion score indicates whether there are any absolute delays in finiteness, by comparing the score of each participant to the cut-off score for that participant's age. Performing at or above criterion score is considered age appropriate. Separate ANOVAs were performed for each criterion: for present tense, for all past tense, and for composite tense including all past and present tenses (Table 14). Gender was added as a covariate.

**Table 14:** **Percent of participants performing at/above or below TEGI criterion for present and past tenses, as well as for composite tense**.

	**Present tense**		**All past tense**		**Composite tense**	
**Group**	**At or above criterion**	**Below criterion**	**At or above criterion**	**Below criterion**	**At or above criterion**	**Below criterion**
**ALI**	38.2	61.8	36.1	63.9	21.6	78.4
**ALI-TD**	80.6	19.4	91.7	8.3	83.3	16.7
**ALN**	80.4	19.6	82.6	17.4	76.1	23.9
**ALN-TD**	97.8	2.2	95.6	4.4	97.8	2.2
**Group differences for at or above criterion**	ALI < ALI-TD^***^, ALN^***^, ALN-TD^***^		ALI < ALI-TD^***^, ALN^***^, ALN-TD^***^		ALI < ALI-TD^***^, ALN^***^, ALN-TD^***^. ALN < ALN-TD |	

*| p < 0.10*,

*** *p < 0.001*.

There was a significant effect of group for the present tense criterion *F*_(3, 156)_ = 15.87, *p* < 0.001 ($\eta_{p}^{2}$ = 0.23), past tense criterion *F*_(3, 158)_ = 20.91, *p* < 0.001 ($\eta_{p}^{2}$ = 0.28), and the composite tense criterion *F*_(3, 159)_ = 30.97, *p* < 0.001 ($\eta_{p}^{2}$ = 0.37). As in other analyses, the effect of gender was not significant. Pairwise comparisons (Bonferroni corrected) indicate that for present, past, and composite tenses criteria, the proportion of the ALI participants performing at or above criterion is significantly lower than all the other groups (all *ps* < 0.001). In case of composite tense, fewer ALN participants performed at or above criterion as compared to ALN-TD controls, and this difference approaches significance (ALN < ALN-TD, *p* = 0.06).

## Discussion

### Overall discussion

Our investigation of finiteness in ASD, the largest to date to include subgroups of children with ASD classified with regard to the presence or absence of language impairment, has revealed two main results (6).

(6) Main Results:
  - Tense/finiteness is severely deficient in children with ALI;
  - Tense/finiteness is not compromised in children with ALN.

For the first time in the literature, we observe that the children with ALI perform significantly lower than both age-matched children with ALN, and much younger TD controls matched on verbal- and nonverbal MA, on all tenses: present, and past regular and irregular. Our youngest control group, the ALI-TD (mean age 6.0 years), shows a 91/92% rate correct (present/past). The ALI group (mean age of 10.6 years) shows a finiteness rate of only 65/68% (present/past). This is not just poor performance, but what one would expect from a very young TD child, at a completely different age level. Furthermore, relative to the composite tense criterion cut off point, only 22% of participants with ALI perform at or above their chronological age cut-off for finiteness vs. 83% of their TD controls. As such, the ALI group's performance is showing a severely delayed finiteness system, which can be called disrupted according to the definition in (3d).

In contrast, our children with ALN performed no differently from their TD controls matched on age, nonverbal and verbal-MA, and grammar: the ALN group (mean age 9.5 years) has an 88/93% finiteness rate which is somewhat (though not significantly) lower than ALN-TD group of the same age (99/97%). As such, the ALN group can be said to have intact finiteness knowledge. However, the criterion analysis for composite tense indicates that only 76% of children with ALN perform at or above their chronological age cut-off for finiteness, which is approaching significant difference from the ALN-TD control group (98%). About 24% of the ALN group does not reach the level of finiteness knowledge indicated by the criterion for their age, showing heterogeneity and variability in the ALN group compared to the TD control group (this will be discussed later in section on Insights from Correlations Analyses).

Our findings on ALN are in line with those reported in Eigsti and Bennetto (2009), Walenski et al. (2014), and Roberts et al. (2004). Eigsti and Bennetto's sample of 10–16 year-old children with autism, high-functioning both in terms of vocabulary and verbal/nonverbal IQ (and in fact higher-functioning than our ALN sample), showed a consistently high performance on all tense morphemes, on a relatively difficult task such as grammaticality judgment (Eigsti and Bennetto, 2009). The findings are also in line with Walenski et al. (2014) whose sample of 7–13 year-old children with high functioning autism and normal verbal/non-verbal IQ showed performance on regular and irregular past tense production that was comparable to their age-matched TD controls. Furthermore, our findings for children with ALI are comparable with Roberts et al. (2004) whose children with ALI also showed substantial difficulties with tense marking compared to age-matched children with ALN and those with borderline language skills.

In short, children with ALN show age-related heterogeneity, but not a systematic deficit in their finiteness scores, while children with ALI show a severely delayed [“disrupted” in the sense that we introduced in (3d) following Rice, 2007] finiteness growth.

In the following sections, we discuss our results in more detail and consider their contribution to our understanding of language impairment in autism, and the phenomenon of the OI stage observed in TD, ASD, and in SLI.

### Is there an extremely extended optional infinitive stage in children with ALI?

An important question, if the study of language in ASD is to be connected to the more general field of language development, is whether children, who show these problems with finiteness, just have some kind of general linguistic deficit, concerning all of language, or whether they are showing the kind of developmental stages that TD children go through. In this paper, we have some of the means to pursue this question with respect to finiteness.

The natural question to ask is, are our participants with autism in the OI stage, a well-known stage that TD English-speaking children grow out of about the age of 4? The stage is sometimes misunderstood to indicate only that the child's grammar allows large amounts of nonfiniteness where finiteness is instead required in the language. As we have seen, this tendency is very strongly observed in our ALI group, and at a very late age. However, the definition of the OI stage (Wexler, 1994, 1998, and others) requires more than the difficulty with finiteness. It also requires a corresponding degree of competence in related areas, which are noted in (7).

(7) Competence required for the OI stage in English:
  - Not having a large amount of omission of non-tense morphemes, specifically knowing enough about aspect and its relation to tense, that a tensed auxiliary followed by a verb requires the verb to be a participle;
  - Knowing the semantic interpretation of the tense morphemes;
  - Knowing the properties of subject-verb agreement.

#### Children with ALI make few errors with -*ing*

First, the OI stage is *not* a stage in which any morpheme (or even any verbal morpheme) is omitted. In particular, the aspectual morpheme *-ing* on present participles is rarely omitted in obligatory contexts during the OI stage in TD and SLI children. Brown (1973) reported very few omissions of *-ing* when finite *be* was produced in TD natural production data at ages even younger than 3 years. Rice and Wexler (1996) found that in spontaneous speech in 5-year-old children with SLI, the rate of -*ing* omission was 8%, comparable with that of the 3-year-old TD children from the same study (10%). Thus, when a progressive tense verb form is used by children, a finite form of the auxiliary *be* is followed by a present participle form of the verb (stem + *-ing*). In other words, this error does not appear to be a representative TD or SLI error.

Children with ALI sometimes used a present or past progressive tense form. This is inappropriate contextually, as we will discuss later in the section on Children with ALI: Inappropriate Response Patterns. These responses, however, allow us to make certain observations.

First, children with ALI produced 48 instances of a finite form of *be* (past or present) followed by a verb. Forty-five of these 48 were present participles; they contained -*ing*. This represents a rate of 93.8% of *-ing* production in obligatory contexts. This gives a small rate of error of 6.2%, compared to the 66–68% rate of the use of a finiteness marker by the ALI group. It seems fair to say that the ALI group does not omit -*ing* in obligatory contexts, which is consistent with the OI stage, in which only special types of morphemes related to tense are omitted.

Secondly, sometimes the present participle appears *without* the auxiliary, a well-known marker of the Optional Infinitive stage where the auxiliary is omitted due to the omission of tense (see Wexler, 1994, 2003, 2004a and, for an empirically adequate theoretical explanation, Schütze, 2004). Of 102 uses of progressives by the ALI group, 46 include an auxiliary, a finiteness rate of about 46%, which is somewhat below the finiteness rate for simple present or simple past tenses. The fact that children with ALI omit auxiliary *be* is another phenomenon consistent with the OI stage.

#### Children with ALI interpret semantic tense correctly

A hallmark of the OI stage is that, although TD children often use a nonfinite form instead of the required tensed form of the verb, they, nevertheless, know the semantic interpretation of the tense morphemes. They do not use a present tense form for past tense or vice-versa (established experimentally for TD children in the OI stage by Schütze and Wexler, 2000). When the context is such that a present tense response is required, and the ALI group produces a tensed response, the tense of the response is *present* 193 times, and *past* 2 times, an error rate of about 1%. When the probe, on the other hand, sets the context such that a past tense is required, and the children with ALI use a tensed response, that response is tensed in *past* 369 times, and *present* 8 times, an error rate of about 2%. Clearly, children with ALI understand the semantic interpretation of the tense value of the present and past morphemes in English and also understand how the contexts make a particular tense appropriate. Not only do the children with ALI understand enough about time and language to achieve such high scores, they understand how to map them onto relevant morphemes. This basic piece of competence in the OI stage is fully realized in children with ALI.

#### Children with ALI know subject-verb agreement

In the OI stage, the basic process of subject-verb agreement is known. Although children often omit a tense/agreement marker in English (in particular here, -*s* or -*ed*), when they *do* use a marker, the subject very strongly tends to agree with it. TD and SLI children very rarely, for example, produce a sentence with -*s* on the verb and a first person subject, e.g., ^*^I goes/we goes (the asterisk indicates that a phrase is not grammatical) (Rice et al., 1995; Harris and Wexler, 1996). Similarly, children in the OI stage in English know the positions of accusative and nominative pronouns; for example, they never place a nominative pronoun in the object position (in Schütze and Wexler, 1996).

Our probes were not specifically set up to test agreement and case, being purposely designed to elicit third person singular subjects and verbs. However, as our results revealed above, we can in general conclude that there were no errors that showed any problems with agreement or case. For example, of the 3 instances of subject *I*, none were followed by a verb with an *-s* inflection. Conversely, whenever a verb with *-s* had an audible subject, it always had a third person pronoun or a noun phrase subject. So far as we can tell from the small number of relevant instances, the ALI group showed the relevant properties of the OI stage.

#### Children with ALI: inappropriate response patterns

Children in the ALI group produced a number of responses that were wrong in the sense that they did not answer the elicitation question. The most frequent response of this type was the use of a noun as a response (34 instances in both present and past tense probes). It is possible that these errors are due to difficulties in attending to the task: Roberts et al. (2004) also found some unusual errors that they attributed to difficulty in understanding the instructions or following the task. This is unlike children with SLI who always answered the prompt with either a finite or a nonfinite verb (e.g., Rice et al., 1995).

The second type of inappropriate response was the use of present progressive participles (with or without auxiliaries) for both the present tense probe (45 instances, 12.4% of total responses) and the past tense probe (57 instances, 8.7% of total responses). The contextual conditions that necessitate the present and past tense responses are somewhat different. In the present tense probe, the lead-in question used a generic with third person and a profession title, “This is a teacher,” followed by the prompt, “Tell me what a teacher does.” This should elicit a generic response, “A teacher teaches.” However, the child uses a progressive form. It is possible that some children with ALI have difficulties understanding the concept of generic/habitual. Further research will have to determine whether this is true. Roberts et al. (2004:441) observe similar errors in their Impaired group of ASD participants.

In the past tense probe, children with ALI provided 21 instances of the *present tense* progressive and only one instance of past progressive (and 35 present participles). This simply seems false instead of inappropriate, or non-answering. Perhaps the child did not understand the intention of the elicitation, which was to point out with simple past that the actor finished the activity described in the picture. However, we know that these children make only about 2% of errors of using a simple present instead of a simple past, suggesting they know how to use present and past tenses. Still, the child, when being asked to describe a completed event, instead describes an on-going event. It seems as if the child is simply ignoring the instructions these 57 times, not attempting to answer in a way appropriate to “finished” but, rather, simply describes the picture that he or she sees. Is it a difficulty in paying attention to the whole context? Or is it something simpler: the child, having difficulties, imitates the tense/aspect of the elicitation sentence, which was in present progressive tense. Alternatively, the fact that some children with ALI have tense and aspect errors may mean that they do not take into account that the probe presented a past action picture given the present progressive situation established by the first picture. This may be a deficit with discourse and not with morphology. This does occur 57 times, and it appears to show a difficulty in integrating all the linguistic information.

In order to argue for deviance in children with ALI with respect to morphosyntax, as in (3b), we would have to find evidence that young TD children do not produce inappropriate responses in an elicitation context, unlike children with ALI. In fact, in case of the use of present progressive tense in contexts eliciting simple present tense, very young TD children (ages 2;5–3;4, mean age 2;11) do make such errors at a rate of 3.6% (Thornton and Rombough, 2015)^12^, which is lower than our ALI group (12.4%). Given the age of the TD participants in Thornton and Rombough (2015), it is quite clear that our observed rates of use of progressive in a simple tense context are at the minimum a sign of disrupted development in children with ALI: their level of cognitive and language functioning is higher, yet their use of progressives in simple tense contexts is much higher than that of 2–3 year old TD children.

Does this suggest that ALI group's inappropriate responses are not deviant? In order to answer this, we have to consider the context of elicitation in Thornton and Rombough's (2015) study, in which, in fact, progressive responses are more felicitous than in our elicitation context. Thus, it is quite plausible that the use of progressive in our contexts by the ALI group is indeed a form of grammatical deviance.

In Thornton and Rombough (2015), TD children were asked to see, for example, if a toy “would fit through the door of a bus” (p. 142). After a couple of affirmative conditions where the toy indeed fit in (in which the experimenter confirmed the observation by an utterance in third person singular simple present tense), the next toy(s) did not fit in, eliciting negation with simple present tense. Most answers were in the adult form using *doesn't* (~40%), followed by such “nontarget” forms of third person singular -*s* as “It's not fit”^13^ or “It not fits.” Two children in particular (out of 25), ages 2;8 and 3;0, account for most of the group's responses in present progressive, using “It's not working,” but notably not ^*^“It's not fitting” (Thornton and Rombough, 2015:153). In particular, one of these children produced many bare stem verbs in affirmative conditions, suggesting that she is firmly in the OI stage.

In order to pragmatically justify an answer like “It's not working,” one needs to do only a tiny bit of accommodation; the child has already accommodated by using *work* instead of *fit*, describing the attempt/goal rather than the action of *fit*. Since some time element is involved in checking out the action, one only has to think about what is on-going. Our judgment is that the progressive is an almost (perhaps wholly) felicitous answer. This is quite different from our elicitation, in which we ask (in the present tense context), “This is a teacher. Tell me what a teacher does.” There is no felicitous response in this context that uses a progressive. We ask for a *generic* or *habitual* answer and receive an activity response. There might be contexts in which an activity response could somehow be accommodated, though we have not thought of one. In this context, an activity response is simply not possible. This use of progressive is a semantic error, not a pragmatic choice. We have suggested that it might be caused by different impairments, not necessarily the lack of understanding of generic or habitual semantics and their relation to syntactic form (though that is possible). In short, an 8.7–12.4% progressive response in our context in the ALI group seems to be clearly a quite deviant answer, in the way that the 3.6% of TD progressive response is not in Thornton and Rombough (2015).

In summary, the above discussion of the relevant OI properties in responses of children with ALI points to an extremely delayed OI stage, in fact “disrupted” in the sense we introduced earlier (see 3d). This is supported not only by their pattern of errors (the nontensed verbs where tensed ones are required, including omissions of auxiliary *be*), but also by their competencies, as described above. Nonetheless, the fact that their responses often involved inappropriate answers, especially with misuse of progressive in generic/habitual contexts, may indicate non-linguistic causes of the errors, and, in fact, deviance from TD (as in 3b). We cannot argue with confidence that the children with ALI are in a pure OI stage. Rather, their performance is consistent with being in the OI stage, but crucially with additional disabilities relevant to morphosyntax.

### Is there an extended optional infinitive stage in children with ALN?

Our results suggest that finiteness is not a serious problem for children with ALN. Their rates of finiteness are significantly higher than for the children with ALI, and not significantly different from the rates for ALN-TD controls. This lack of a serious deficit in finiteness indicates that the children with ALN are not in the OI stage at their chronological age.

Given that rates of finiteness are so high in the ALN group, we would not expect errors that are characteristic of the OI stage. For reasons of completeness, we note that the ALN group made no errors of interpretation on past and present tense, never omitted *-ing* when required after an auxiliary, made no subject-verb agreement errors or case errors (although, like the children with ALI, they had limited situations in which the latter errors could occur). Of eight inappropriate uses of the progressive tense by children with ALN, five include omission of the auxiliary. This omission would be consistent with the OI stage, but this number is too small to be interpreted in any meaningful way. The lack of -*ing* errors is consistent with Eigsti and Bennetto's (2009) findings that children with high functioning autism easily recognize the omission of -*ing* and with Tovar et al. (2015) who find, using intermodal preferential looking methodology, that 4-year-old children with ASD, functioning in the borderline range, show some comprehension of the difference between progressive and simple tenses. The use of progressives instead of simple tense in our ALN group was extremely low, indicating no particular difficulty in integrating information from a few sentences to achieve the correct response given the context. While the ALI group responded inappropriately 34 times with a noun, there was only one such instance for the ALN group.

We conclude that the ALN group is not in the Extended OI stage and has the grammatical capacity that goes beyond that stage, on par with their ALN-TD controls. The ALN group's competent performance on our finiteness tasks indicates no overlap with SLI whatsoever. A future study should investigate finiteness in very young children with ALN, who would be expected to be in the OI stage in virtue of their young age.

### Optional infinitives and null subjects in ALI and ALN

A well-known phenomenon in TD children (Hyams, 1986, and many papers since) is the tendency of young children to omit subjects of sentences. By now there has emerged reliable evidence concerning some of the major properties of these null-subjects, and how they relate to the OI stage.

First, there is a much larger tendency to omit the subject if the child produces a nonfinite (untensed) verb. Wexler (e.g., Wexler, 1994; Bromberg and Wexler, 1995:222) argued that this was because verbs without tense can license null-subjects in the adult language, and the child was simply in agreement with this grammatical fact. The Agreement Tense Omission Model (ATOM) of Schütze and Wexler (1996) allows tense to be omitted from the structure, thus also allowing a null-subject for untensed verbs. Once the child produces a nonfinite verb, a null-subject is grammatically appropriate.

Second, in young TD children there are still many instances of null-subjects of finite verbs, a result that cannot be explained by resorting to grammatical possibilities when the verb is nonfinite. We will discuss why this possibility exists after discussing the results concerning null-subjects in ASD.

As Table 8 shows, both ALI and ALN groups produced large numbers of null-subject utterances, in both present and past contexts. We will return to the question of why there is no significant difference between children with ALI and ALN in proportion of utterances with null-subjects.

Let us go into more detail starting with the ALI group. Table 9 shows that the ALI group produces a greater proportion of null-subjects when the verb is untensed than when it is tensed, for both tenses. This pattern is exactly what is found in null-subject production during the OI stage in TD children, and is well understood. The pattern provides further indication that the children with ALI are in the OI stage of grammatical development, and that their responses are based to some extent on their grammatical knowledge. Nonfinite verbs license null-subjects grammatically in adult and child language.

Moreover, the pattern provides evidence concerning the possibility that the children with ALI are omitting subjects because they have memory limitations: it may be difficult for children to produce a long sentence that includes a subject. This is an old idea in non-grammatical approaches to child null-subjects that are not grammatical in the adult language. Suppose, as e.g., Bloom (1990) argues, that the reason children drop subjects is because of limited working memory (shown to be incorrect by Hyams and Wexler, 1993 for TD children). The idea is that a longer verb phrase leads to more null subjects. The expectation then is that children will omit more subjects with finite verbs, since those verbs are longer (bare stem + inflection morpheme). In fact, we find the opposite result. This leads us to believe that the “more null subjects with nonfinite verbs” pattern, which holds of both our ASD groups and TD children in the literature, is induced by the grammatical properties of the underlying language development mechanism, as is generally accepted in TD. Even the ALI group is seen to have a grammatical system that is the cause of much behavior, even when the system is quite immature.

The children with ALN also produce particularly large rates of null-subjects with nonfinite verbs, reaching 93% for past tense and 73% for present tense probes. Possibly these large proportions are a consequence of the grammatical possibility of null-subjects with nonfinite verbs (there are relatively few of these for the ALN group, as we have seen).

It is also possible that some of these responses are due to the possibility of potential, almost grammatical, replies in our experiment. The past tense probe showed a picture in which e.g., a girl is skating, then one in which the action was completed, and the child was told, “Here she is done. Tell me what she did.” One can imagine an almost grammatical answer, “skate.” One possibility is that the answer is a reduced form of “What she did was skate,” with everything but *skate* elided, given its recoverability. The answer cannot be ^*^she skate; it must have a null-subject. The children with ALN are only taking advantage of this nonfinite possibility 29 times (vs. 256 finite responses), but when they do, 27 of the responses have null-subjects. If this explanation is on the right track, we can see that the ALN group's responses in the past tense are again strongly consistent with being out of the OI stage. The children with ALI, however, produce fewer null-subjects in the past tense than in the present tense. It might very well be that they are not particularly taking advantage of the reduced nonfinite response, but are simply trying to indicate a simple subject and verb and putting the verb in the untensed form as part of the OI stage.

In case of the present tense probe, the elicitation says e.g., “This is a teacher. Tell me what a teacher does.” It also seems possible that there is a potential response like, “What a teacher does is teach,” reduced to *teach*, again an untensed form of the verb. The ALN group produces 73% of its nonfinite verbs with a null-subject, possibly in accordance with this possibility. The ALI group produces 68% null-subjects in this case, probably again indicating a null-subject licensed by a nonfinite verb.

We also have to allow for the possibility that the greater proportion of null-subjects is due to the fact that our elicitation provided a strong common ground (topic) and a question about what the common ground *does/did*, which allows for one way of answering that elides the common ground/topic and uses a nonfinite verb. Determining with more certainty why the two groups are producing the greater proportion of null-subjects with nonfinite verbs (whether it is due to the general licensing of null-subjects with finite verbs, or whether it is due to the strategy that we have indicated that works for this particular elicitation) requires further research. The elicitation task of TEGI does not allow us to disambiguate between these two possibilities, which, as a reviewer suggests, may underestimate children's grammatical knowledge. The results on null-subjects that we have attained, however, do argue that the responses of both the ALI and ALN groups are guided by the grammatical structures that they have, rather than by any kind of simple memory limitation.

We are left with the issue of null-subjects of tensed verbs, a much-discussed issue in TD. We will adopt the model in Wexler (2013), in which sentences in which both the subject and the predicate are discourse-old are grammatically Tense Phrases (TP's) rather than Complementizer Phrases (CP's) as argued by Mikkelsen (2015) for Danish. Thus, a subject in such a sentence is the specifier of a root, which may be omitted (Rizzi, 2006) because there is no higher projection that allows its spell-out. Wexler (2013) argued that young children often take sentences to be discourse-old even when they are not, thus taking structures to be TP's too often, leading to subjects that are specifiers of a root (which may be omitted), leading to null-subjects of finite verbs. In simple terms, the ultimate explanation for children's use of null-subjects with finite verbs is their immature knowledge of information structure. Once this plays a role, the child's syntactic system will induce the possibility of a null-subject. Thus the combination of an immature knowledge of information structure and a more mature grammatical system will lead to the possibility of null-subjects (Wexler, 2013).

The null-subjects of finite verbs in both the ALI and ALN groups may follow from this lack of knowledge of information structure. It might very well be that the kind of defining issues for ASD, e.g., issues related to Theory of Mind, may be enough to cause the relevant difficulties with information structure (which is an interface module, relating syntax to pragmatic/discourse conditions) in both the ALI and ALN groups although their ages would not be consistent with this difficulty in TD. The null-subjects of finite verbs at this late age (~9–10 years) may very well be a sign of autism, whether grammatically impaired (ALI) or grammatically not impaired (ALN). The model of autism that we are working with, and the model of null-subjects and grammatical development more generally that we are working with, predict this particular difficulty for both groups of children with ASD. Further research could be directed toward investigating the consequences of these considerations and toward a more focused attempt to study null-subjects with finite verbs.

To compare, 4-year-olds with SLI, who used 33% nonfinite verbs in their spontaneous production, only showed 16% null subjects with nonfinite verbs, and 2% null subjects with finite verbs; TD children aged 4 and higher showed no null subjects (Schaeffer et al., 2002). The SLI rates are much lower than either of our much older ASD groups, suggesting that information structure is not impaired in SLI. This is one more piece of evidence that ALI is not ASD + SLI, which will be discussed in more detail below.

To recap, the fact that both ASD groups differentiate between null-subjects-with-finite-verbs and null-subject-with-nonfinite-verbs suggests, once again, that children with ASD are not simply omitting surface morphemes or words, but are actually producing different linguistic derivations for nonfinite vs. finite verbs, showing a somewhat functioning language system. On one explanation, children with ASD seem to be exhibiting more difficulties with the knowledge of information structure, in particular with the determination of whether or not subjects and predicates are discourse-old.

### Insights from correlations analyses

#### Finiteness, chronological age, and standardized measures of language and IQ

What other factors, linguistic and non-linguistic, influence the acquisition of finiteness? What can we discover about the relationship of grammar and other cognitive abilities by focusing on the acquisition of different aspects of finiteness by typically and atypically developing populations?

According to Wexler (1996, among many others), finiteness in TD and SLI children grows over time according to some internally set maturational schedule, which may not be directly related to other cognitive abilities. In Rice et al. (1998), the best predictor of finiteness growth in TD and SLI was age. Receptive vocabulary (PPVT), nonverbal reasoning abilities and mother's education were not significant predictors. This is quite telling, considering the well-known finding that a child's vocabulary is predicted by mother's education and is a measure of environmental input (cf. Rice et al., 1998:1418).

Our correlations analyses provide evidence that the ALI group does not have a language system that functions akin to that of the TD children and the children with SLI of Rice et al. (1998): finiteness in our ALI group is not dependent on age. In fact, overall language abilities (expressive and receptive vocabulary, receptive grammar) and NVIQ strongly correlated with finiteness deficits in the ALI group. This is partly in line with the results of Roberts et al. (2004) who found that past tense performance of their ASD group correlates with age, verbal and nonverbal IQ, and receptive vocabulary scores. Furthermore, we find the same difference as Roberts et al. for present tense: all measures except NVIQ seem to play a role. Our findings also agree with Eigsti and Bennetto (2009) who found significant or near significant correlations between their high-functioning group's scores on grammaticality judgements and expressive vocabulary, verbal and nonverbal IQ. On the other hand, our results for ALI contrast with those of Botting and Conti-Ramsden (2003) who found that NVIQ does not correlate with past tense knowledge in ASD. In this expanded respect (not related to grammatical constructions, but to developmental pattern), knowledge of finiteness, or at least the mechanisms underlying it, is deviant in children with ALI compared to SLI and TD children^14^.

In the ALN group, on the other hand, finiteness strongly correlates with chronological age only, and, as in TD and SLI children from Rice et al. (1998), standardized test scores rarely correlated with tense, indicating a functioning maturing language system with respect to finiteness. Evidently, some younger children with ALN are showing weaker finiteness knowledge than older children with ALN, thereby introducing some heterogeneity in the ALN group as evidenced by the criterion analysis (see the section Overall Discussion). Thus TD, SLI, and ALN pattern together in showing age as a causative factor in development, whereas ALI stands apart, a kind of (non-constructional) deviance.

The finding of a lack of correlation for the ALI group between a child's age and composite tense score is striking, but should be taken with some caution. Perhaps some other variable affected whether a child with ALI gets into the study, a variable that correlates with age. A longitudinal study, in the manner of Rice et al. (1998), could shed more light on the issue of whether children with ALI improve their scores on composite tense as they age.

#### Finiteness and ADOS and ADI-R

Before discussing our results in this section, it is necessary to look into the similarities and the differences between ADOS and ADI-R tests, which are complementary measures of the ASD symptomatology.

ADI-R is a structured interview of a parent or a caregiver, with questions focusing on a child's current behaviors (Current Algorithm) as well as behaviors observed at the most abnormal stage of the child's development so far, usually 4–5 years old (Diagnostic Algorithm). ADI-R assesses abnormalities in the domains of social interaction, communication, and behavior. The measure notes whether a child is verbally fluent (able to produce phrases of three or more words).

ADOS is a structured series of activities and interactions between an evaluator and a child, providing a snapshot of the child's behavior at the time of testing. ADOS measures a similar range of social and communicative behaviors to ADI-R. The test has different modules depending on whether a child is verbally fluent (sentences with multiple clauses) or not (just three-word phrases).

Neither ADOS nor ADI-R directly addresses any specific grammar skills.

Our correlations between finiteness rates and scores from ADOS and ADI-R measures indicate distinct profiles for ALN and ALI groups. In children with ALI, finiteness, especially composite tense, is strongly associated with scores from all ADI-R Current Algorithm domains. Correlations with ADOS measures were less robust, and nonexistent for composite tense. Furthermore, there were no correlations of tense with any of the ADI-R Diagnostic Algorithm scores. The latter observation suggests that estimation of early dis/abilities does not correlate with finiteness dis/abilities at a later age.

The ALN group, on the other hand, showed only a few associations with ADOS Communication and with ADI-R Diagnostic Algorithm Social Interaction scores. Composite tense did not associate with any of the tests for ASD. These findings are in part comparable to Lindgren et al. (2009), who found that ALN and ALI groups' total language scores on measures of morphology, syntax, semantics and verbal memory from a standardized test, CELF-3, did not correlate with ADOS and ADI-R scores. In contrast, Eigsti and Bennetto (2009) found significant correlations between performance on a grammaticality judgment task of their high-functioning group (who are likely similar to our ALN group) and ADOS measures of Communication and Social Interaction (but not Repetitive Behaviors).

If ADOS and ADI-R are largely measuring the same aspects of ASD symptomatology, why do we find these differences in correlations in ALI? Lack of correlations with ADOS could be explained by the fact that we tested our participants on average a year after they were tested on ADOS and ADI-R. This explanation, however, cannot account for our correlations of the ADI-R Current Algorithm scores and finiteness, which ought to show the same differences in behavior with time. Therefore, an explanation may stem from the differences between ADOS and ADI-R measures. Could it be that parental observations of current daily behavior are in some sense more relevant to the knowledge of finiteness than a clinical interactive observation that lasts an hour or so? It is unlikely that parents estimate their children's verbal fluency by awareness of whether their children produce non/finite verbs. Rather, it may be possible that finiteness is a precursor to overall fluency which parents are sensitive to.

Putting our correlations results together, it seems that in children with ALI, their low overall verbal and nonverbal IQ and their receptive language abilities, as well as the severity of their current symptoms of autism, correlate strongly with their rates of finiteness, which is very different from what we observe in our ALN sample, and in children with SLI and their TD controls studied by Rice et al. (1998) for whom it is primarily chronological age that correlates with finiteness. As such, we can say that the mechanisms underlying grammar abilities are different in children with ALI and with ALN.

### Is ALI the same as ASD + SLI?

Here, we compare our results on finiteness in our children with ALI with those of children with SLI and younger TD children from Rice et al. (1998). The notable difference, of course, is that their SLI group was not impaired on NVIQ (following the selection criteria for SLI) whereas our ALI group was. The standard scores of our ALI group and their 5-year-old SLI group (SLI-5) are comparable on receptive vocabulary (though our ALI group is on average older than the SLI group). However, on measures of receptive grammar, our ALI group's standard scores are substantially lower than those of SLI-5.

In terms of performance on rates of finiteness, our ALI group (aged 10.6) is much better than SLI-5 (twice as high, in fact). Our ALI group is most comparable to Rice et al.'s participants with SLI at ages 6.0 or 6.5, and is lower than their TD group at age 3.5 (but better than the 3-year-olds from the same study). In our participants, there are much greater standard deviations, suggesting a greater variability in ASD than in SLI.

Although children with ALI and SLI may show some similar levels of finiteness, albeit at different ages and levels of general language and cognitive abilities, the overall differences between groups are very great, and thus we hesitate to state that there are similarities between ASD and SLI. Furthermore, we described some kinds of errors that the children with ALI make that the children with SLI are not known to make (see the section Children with ALI: Inappropriate Response Patterns). The children with SLI are in an extended OI stage, showing the same morphosyntactic deficits and competencies as found in young TD children; the children with ALI cannot be said to be in a pure extended OI stage because they show evidence for some patterns that are not found in the OI stage.

Furthermore, there is a conceptual unclarity in what is meant by the formula: ALI = ASD + SLI. Since all researchers are ultimately interested in the etiology (including genetics) of these syndromes, the simplest assumption would be that the syndrome ASD (having no grammatical deficit by itself) is sometimes independently inherited with the syndrome SLI. Such a proposal makes grammatical deficits simply not intrinsic in any way to ASD, with grammatical language impairment in ASD being inherited by chance. Let us call this the *Independent Inheritance* proposal.

Given that the rate of SLI in children is about 7% (Tomblin et al., 1997), if ASD and SLI are independently inherited, there should be a rate of 7% of grammatical language impairment in all of ASD. We are unaware of epidemiological studies that measure the relative rates of ALI and ALN. Our data can give us a measure, thus allowing us to test whether this prediction is true. We selected our 83 ASD participants without any regard as to whether they were grammatically impaired or not, and tested to categorize them as 46 ALN and 37 ALI participants. The numbers of children with ALI are around 45% of our total ASD sample. This is comparable to other studies of ALI: e.g., the study by Roberts et al. (2004) had 19 children with ALI out of 62 participants (just under a third of all the participants), while Kjelgaard and Tager-Flusberg (2001) had 50 out of 82 (just under two thirds of all participants)^15^. These rates of ALI are much greater than the expected 7% from the *Independent Inheritance* assumption. Thus, we argue that biologically, ALI is *not* an independent chance merger of ASD and SLI in the same child; that is, ALI is not ASD co-morbid with SLI (contrary to, e.g., Tager-Flusberg, 2015)^16^.

It is likely that the disorder of ALI (unlike high-functioning autism or ALN) itself causes a range of deficits in the development of different aspects of language, just as other disorders, such as Down syndrome (DS) and Williams syndrome (WS), do. There are examples in the literature for such aspects of complex language as binding dependencies (Perovic et al., 2013a,b, for ASD; Perovic and Wexler, 2007, for WS; Perovic, 2006, for DS) or passive constructions (for ASD: Perovic et al., 2007, for English, and Durrleman et al., 2016, for French; Perovic and Wexler, 2010, for WS; Ring and Clahsen, 2005a, for DS). The fact that, e.g., omission of verbal inflection in ASD is showing similar patterns across disorders (also for DS: Ring and Clahsen, 2005b; and WS: Peregrine et al., 2006), as well as some similarities to TD (though with some notable differences), is simply an indication that the starting point of language acquisition, the innate genetically-guided language learning system, is the same in all disorders and TD, but is affected differently by the respective disorders. We have argued in various publications that neurodevelopmental impairments seem to allow grammar to develop up to a certain point in a maturationally (biologically) determined way, such that in a particular impairment, the child reaches only a certain level (e.g., Perovic et al., 2013a,b, for ASD; Rice et al., 2009a, for SLI). This was observed in other domains as well: Landau (2012:83) suggests that for spatial representation, “People with WS appear to hit the functional level of a 4- or 6-year-old normal child, but do not grow further.” Thus, we would expect finiteness, a biologically determined slow development, to be subject to impairment, as suggested in Wexler (1996). The question of how equivalent ALI and SLI children are grammatically in general will depend on investigation of more complex grammatical structures, an investigation that is under way, but that we will not discuss here.

## Conclusions

Our extensive study of finiteness and morphosyntax in two large groups of children with autism and their matched TD controls shows different morphosyntactic abilities relative to the presence or absence of a general language impairment.

Our ALI group shows extensive deficits with finiteness, which are not only large quantitatively, but are also not construction specific, appearing in simple present and past tenses, as well as with auxiliary omission. These difficulties in children with ALI, along with their morphosyntactic competence, are similar to what is observed in very young TD children (much younger than the ALI-TD controls in our study) and indicate disrupted development. The maturational mechanisms underlying the knowledge of finiteness, however, are likely different between those with ALI and those with TD or SLI: autistic symptomatology and overall cognitive and language abilities strongly correlate with finiteness in ALI whereas age does not, indicating a deviant development. Further evidence of deviance comes from the ALI group's use of progressive in habitual/generic contexts. All this suggests that our ALI group is both deviant and disrupted in its knowledge of tense marking. The children with ALI may show some properties, both deficits and competencies, of the OI stage, but they have patterns that go beyond the observed TD or SLI profiles.

On the other hand, there is somewhat slower development of finiteness in children with ALN than their chronological age warrants, but it is still comparable to their TD controls. Furthermore, their knowledge shows evidence of a maturational language learning mechanism, not influenced by autistic symptomatology. However, information structure in ALN shows some deficits, similar to very young TD children and the impaired ALI group. This is striking because information structure deficits should be expected to apply to ASD in general, given the nature of ASD (especially difficulties with pragmatics). Thus children with ALN have pragmatic (in particular information structure, which depends on discourse) difficulties, but not grammatical difficulties, in contrast to children with ALI, who have disrupted tense-marking capacities (in addition to the difficulties with information structure).

It is possible that in all children, the same genetically coded language learning mechanism, called “Universal Grammar” by linguists, is present, and that gives us the ASD and SLI performance consistent with the OI stage of TD children. The genetic deficits of neurodevelopmental disorders then work to limit different aspects of language acquisition, whether grammar or information structure, differently depending on the disorder and its severity.

Following an original suggestion by Wexler (1996), finiteness has already been used as a biomarker to guide studies of genetics of language: in twin behavioral studies (in TD children, Ganger, 1998, and Ganger et al., 1998; and in children with SLI, Bishop et al., 2005), and in genetic linkage studies of families with SLI (Falcaro et al., 2008; Rice et al., 2009b). In comprehensive reviews, Rice (2012, 2013) integrated the findings about trajectories of language development in SLI with their possible genetic bases.

In autism, finiteness has not yet been used as a biomarker. It is possible that deficits in finiteness can not only assist in distinguishing children with morphosyntactic language impairments or delays within autism subgroups, but also guide genetic studies of language. For example, some genes that are regulated by *FOXP2*, a transcription factor involved in a familial speech-language disorder, have been implicated in language deficits (Graham and Fisher, 2015). One such gene is *CNTNAP2*, which is associated with a non-word repetition deficit in SLI (Vernes et al., 2008), with delay in producing a first word in males with autism (Alarcón et al., 2002, 2008), and with level of language-related behavior at age 2 in children from an unselected sample of the general population (Whitehouse et al., 2011). Thus, it is possible to suggest that the same genetics may underlie different aspects of language development. It will be interesting to see how and whether knowledge of finiteness in ASD associates with genetic variants.

Future studies may address other specific markers associated with Tense, and they should also address other aspects of language that are argued to be deficient in children due to the same computational mechanism that limits finiteness. In particular, the Unique Checking Constraint theory of the OI stage predicts that in some (but not all) languages, clitic pronouns should be omitted (Wexler, 1998, 2004b, 2014, among others). The theory predicts that TD children (and thus children with SLI) will not omit object clitics in Greek, and that was confirmed for TD children by Tsakali and Wexler (2003) and for children with SLI by Manika et al. (2011). One such study has already been done in Greek for 6-year-old children with high-functioning autism (Terzi et al., 2016), who show lower clitic production than age and receptive vocabulary matched TD controls, which indicates deviance. In this way, the studies in the field of grammar in autism will advance to the level of the study of the theory of developmental mechanisms, rather than individual constructions, paralleling advances in the study of typical development.

## Author contributions

KW and AP conceived the study. AP and NM contributed substantially to data collection. NM contributed substantially to transcribing, analyzing and interpreting the data as well as writing the initial draft of the manuscript, with all authors doing some of the writing of some sections. All authors contributed substantially to editing the final versions of the manuscript. All authors have agreed to be accountable for the content of the manuscript.

## Funding

NM was supported in part by the Singleton Fellows Graduate Fellowship from the Department of Brain and Cognitive Sciences at the Massachusetts Institute of Technology (MIT), and the Simons Postdoctoral Fellowship from the Simons Initiative on Autism and the Brain at MIT. AP was supported by the Brain Development and Disorders Project (BDDP) Postdoctoral Award from MIT. This research was also supported by the Anne and Paul Marcus Family Foundation, and the Brain Infrastructure Grant Program to the Department of Brain and Cognitive Sciences, MIT, from the Simons Initiative on Autism.

### Conflict of interest statement

KW is a co-author on the Test for Early Grammatical Impairment (Rice and Wexler, 2001) which was used for testing the participants in the present study. Other than that, the authors declare that the research was conducted in the absence of any commercial or financial relationships that could be construed as a potential conflict of interest.

## Footnotes

^1^The terms “tense marking” and “finiteness” will be used interchangeably throughout the paper to refer to the phenomenon that verbs in most main clauses in adult sentences must be marked for tense, which makes them finite.

^2^A further 29 children were recruited, but had to be excluded for various reasons (detailed here and in the footnotes to follow). Of these, 11 participants with ASD were excluded due to their inability to complete the battery.

^3^For 15 participants, confirmed diagnoses were not available due to difficulties with data sharing. On average, our study was performed with ASD participants 1.14 years (*SD* = 1.06) after the administration of ADOS and ADI-R.

^4^Three participants did not meet the criteria of either ADOS or ADI-R and were thus excluded despite their clinical diagnoses of Pervasive Developmental Disorder—Not Otherwise Specified (PDD-NOS).

^5^This classification left us with 14 “borderline” participants who scored at or above the 10th percentile on both vocabulary tests, but below the 10th percentile on TROG-2, with SS of 72–79, and thus could not be classified into either ALN or ALI (cf. Roberts et al., 2004). Their small number, 14, compared to large numbers in the ALN (*n* = 46) and ALI (*n* = 37) groups precluded us from forming a separate group; thus, it was decided to exclude these children from the current analysis. Of note, this group's performance on finiteness and other measures was in between that of the ALI and ALN groups.

^6^Seventeen participants with ALN and 19 participants with ALI were included in the sample in Perovic et al. (2013b)'s study of binding.

^7^For one ALI participant and one ALN participant, the KBIT Matrices scores were unavailable, leaving them with no matched TD controls.

^8^When we report the data that go beyond the major planned measure of this paper, that is finiteness rates, we compare ALN and ALI. We do not report results for TD. This is because in moving a laboratory, we lost the original data sheets for the TD participants. We already had their finiteness measures, per participant, but had not yet analyzed the extra measures (like null-subjects) that we calculated. Since we have the ALN measures, which show very little error on many of these responses, we can compare them to ALI.

^9^A reviewer made the interesting suggestion that instead of being instances of -*ing* omission, the three sentences could be instances of tense marking in the auxiliary. This would be plausible, that is, grammatical for English (though pragmatically odd) if the auxiliary was a finite form of *do*, as in *he does splash*. With a form of *be*, the sentences are ungrammatical. If the child believes that such forms are grammatical, then the sentences are at least as deviant (for English) as the omission of *-ing*. We take *-ing* omission to be a descriptive term; we do not believe that our data are capable of determining the grammar of *-ing* omission. A study that focused on that question would be of interest although the relevant percentages are small in young TD children.

^10^For the remaining participants, no subjects were recorded on the answer sheets, likely because no subjects were produced. However, to be conservative, the responses from these participants were excluded from these counts.

^11^Note that the significance levels for our correlation analyses (here and in the next section) were not Bonferroni corrected; we will use them only in trying to observe particular patterns that might suggest specific hypotheses and further studies.

^12^We are grateful to a reviewer for this clarification of disruption and deviance, as well as for pointing out Thornton and Rombough's work to us.

^13^Utterances in this form, with a *be* auxiliary and a bare verb, can be considered to show -*ing* omission. The rate of these phrases in Thornton and Rombough (2015) is very small, 3.2% of total responses, and is consistent with prior observations for -*ing* omission.

^14^We are grateful to a reviewer for clarifying this aspect of deviance.

^15^It is important to also note that few studies investigate linguistic abilities in large numbers of children with ALI although there are regular references to ALI in the literature (e.g., Bishop et al., 2016).

^16^A weaker formulation of ALI = ASD + SLI is possible. It might be proposed that (for some reason) the genes underlying ASD and SLI have a strong tendency to be inherited together, so that the chance of the co-occurrence of inheriting SLI if a child has ASD is much larger than the chance of inheriting SLI if a child does not have ASD. The statistical argument above does not count against such a formulation. Questions of grammatical deviance and rates of finiteness, however, are still relevant, counting against the hypothesis.

## Abbreviations

ALI: autism language impaired
ALI-TD: autism language impaired—typically developing controls
ALN: autism language normal
ALN-TD: autism language normal—typically developing controls
ASD: autism spectrum disorders
ATOM: Agreement Tense Omission Model
CA: chronological age
CELF: Clinical Evaluation of Language Fundamentals
DS: Down syndrome
EOI: extended optional infinitive stage
KBIT: Kaufman Brief Intelligence Test
MA: mental age
MLU: mean length of utterance
n.s.: nonsignificant
NVIQ: nonverbal intelligence quotient
OI: optional infinitive
PPVT: Peabody Picture Vocabulary Test
RS: raw score
SLI: specific language impairment
SS: standard score
TD: typical development or typically developing
TEGI: Rice/Wexler Test of Early Grammatical Impairment
TROG: Test of Reception of Grammar
WS: Williams syndrome.
